# Marital Status and Survival in Patients Diagnosed with Melanoma

**DOI:** 10.1155/2020/2485401

**Published:** 2020-01-30

**Authors:** J. A. Maas, A. J. Monreal, E. L. Diaz, G. Castro, P. Rodriguez de la Vega, M. Varella

**Affiliations:** ^1^Hazelrig-Salter Radiation Oncology Center, University of Alabama at Birmingham, Department of Radiation Oncology, 1719 6th Avenue South, Birmingham, AL 35294, USA; ^2^Herbert Wertheim College of Medicine, Florida International University, Department of Medical and Population Health Sciences Research, Modesto A. Maidique Campus (MMC), 11200 S.W. 8th Street, Office # AHC1-340, Miami, FL 33199, USA

## Abstract

**Introduction:**

Previous research suggests the presence of a spouse may considerably affect melanoma detection rates through more frequent examinations, better access to healthcare, and improved social support. Yet, the role of marital status on melanoma survival is currently unknown. The aim of this study is to assess whether marital status is associated with survival following melanoma diagnosis.

**Methods:**

We performed secondary analysis of data from all participants of the Florida Cancer Data System (FCDS) and included adult melanoma patients diagnosed between 2001 and 2009 with follow-up information available until 2015. Marital status was categorized as single, married, divorced, or widowed. The primary outcome was survival interval after melanoma diagnosis, which was assessed according to the time from the date of diagnosis to the time of death or last contact. Cox proportional hazard models were used to assess the independent association between marital status and survival.

**Results:**

We assessed data from 36,578 melanoma patients. Married patients were significantly more likely to survive than single patients (Hazard ratio (HR) = 0.65; 99% Confidence Interval (CI): 0.57–0.74; *P* < 0.001) after adjusting for age, sex, race, ethnicity, geographic location, insurance status, tobacco use, primary site, stage, and histology. There was no evidence of effect modification by gender (*P*=0.189).

**Conclusions:**

Married patients, including both men and women, had a 35% reduction in the risk of death after melanoma diagnosis compared with single patients, and mechanisms independent of earlier detection, such as social support, may play a role in survival in patients with melanoma.

## 1. Background

Melanoma is the sixth most common cancer in the United States (US) as well as the most life-threatening form of skin cancer [1]. Despite improved case mortality in the US, overall deaths due to melanoma have increased because of an increase in melanoma incidence [2]. The annual burden of invasive melanoma in the US from 2004 to 2006 was found to include over 45,000 invasive melanomas diagnosed annually, corresponding to a rate of 19 cases per 100,000 persons and resulting in 8,246 deaths [3]. However, only ten years later, the American Cancer Society estimated 76,380 new invasive melanoma diagnoses and 10,130 invasive melanoma deaths in the US in 2016 [1]. Additionally, the average annual treatment costs for melanoma from 2007 to 2011 were $3.3 billion [4].

Prevention and early detection have been shown to have substantially protective effects in curtailing melanoma cancer burden among patients [5, 6]. More recently, the presence of a spouse has been described as a factor that could considerably affect skin cancer prevention as it has been linked to detection of melanoma at an earlier stage [5, 7, 8] and decreased melanoma thickness at diagnosis [9, 10]. Having a partner has also been linked to better access to healthcare and social support [5, 11]. While married patients in the US were found to have significantly lower risk of undertreatment, presentation with metastatic cancer, and death when diagnosed with other common types of cancers, specific information on melanoma is lacking [12].

Florida has the second highest incidence and overall melanoma cancer burden in the US [13, 14]. Moreover, the State of Florida is unique in its demographic and cultural characteristics; for example, 24.5% of the Florida population is comprised of Hispanics or Latinos [15]. Findings regarding marital status and melanoma in Florida might provide internationally generalizable insight into the medical-social relationship of patients suffering from melanoma. As such, we aim to assess the association between marital status and survival in melanoma patients registered in the Florida Cancer Data System from 2001 to 2009.

## 2. Methods

### 2.1. Study Setting

We conducted secondary analysis of data collected from the Florida Cancer Data System (FCDS), a population-based, statewide cancer registry [13]. Briefly, the FCDS is primarily a hospital-based reporting system that collects information on incident cancer cases in Florida. All health-care facilities licensed under Florida statute 395, freestanding radiation therapy centers, ambulatory surgical centers, licensed medical practitioners, and/or any laboratories are required to report cancer cases to the FCDS.

### 2.2. Study Participants

We analyzed data from all adult patients, as recorded in the FCDS, which reported an ICD-O-3 code of melanoma (*International Classification of Diseases for Oncology*, Third Edition (ICD-O-3), codes C440-C449, types 8720–8780) diagnosed from 2001 to 2009. Patient follow-up information was available from the FCDS until the year 2015. Survival status is based on information from the State of Florida Bureau of Vital Statistics.

### 2.3. Main Variables

The independent variable was marital status at the time of diagnosis (categorized as married, single, divorced, or widowed) as reported to the FCDS. Patients with unknown (*N* = 5660) and separated (*N* = 220) marital status were excluded. The main dependent variable was survival interval after melanoma diagnosis. Survival time was obtained by measuring the interval between the time of cancer diagnosis and the time of death or last recorded contact. The event (death) was considered present if death occurred at the end of follow-up time. Patients were considered free of event at the end of follow-up in 2015 or if they were alive at the time last seen (administratively censored). Confounding variables assessed included patient's age, sex, health insurance status, race, ethnicity, geographical area, tobacco use, tumor histology, cancer stage at time of diagnosis, and anatomical tumor site. Cancer stage at diagnosis was categorized using the available SEER staging system: in situ, localized, regional by direct extension, regional lymph nodes, and distant. SEER stage in situ corresponds to AJCC stage 0, SEER stage localized corresponds to AJCC stages I and II, SEER stages regional by direct extension and regional lymph nodes correspond to AJCC stage III, and SEER stage distant corresponds to AJCC stage IV. SEER stages in situ and localized (corresponding to AJCC stages 0, I, and II) were grouped together as the early stage; SEER stages regional by direct extension, regional lymph nodes, and distant (corresponding to AJCC stages III and IV) were grouped together as the late stage. Less than 10% of data were missing for each variable except for tobacco use.

### 2.4. Analytical Plan

Description of the study participants and assessment of patterns of missing data and outliers were performed. Subsequently, identification of potential confounders to be accounted for in the multivariable models was performed through bivariate analysis using survival status at the end of follow-up (dead or alive) as the outcome. Survival probabilities were calculated and presented using the Kaplan–Meier methods, and statistical significance was assessed using the log-rank test. Lastly, different multivariate Cox proportional hazard regression models were tested to assess independent associations between marital status and survival time. SPSS v.20 software [16] was used for all analysis, and statistical significance was considered for *p* values below 0.01 for a 2-tailed test. Revision by The Florida International University Institutional Review Board considered the study to be nonhuman subject research.

## 3. Results

In total, 36,578 patients were included in the study. The average age of participants was 62.5 years at diagnosis (standard deviation (SD) = 16.2) (Table 1). A total of 25,438 patients were married (69%). Most melanoma patients were white (99%), non-Hispanic (97%), and male (59%). Of the patients with staging information available, most patients were diagnosed at an early stage (88%). Only about 25% had the diagnosis of one of the main histologic subtypes of melanoma (superficial spreading, nodular, lentigo maligna, and acral lentiginous); most of the remaining cases had unclear histology as they were diagnosed as melanoma, not otherwise specified. The characteristics of the participants varied according to the marital status (Table 1). Widows were older than married and divorced patients, while single patients were younger. A larger percentage of married individuals (64%) were male, while fewer widowed patients (33%) tended to be male. Both widowed and married individuals were more likely to be insured, while single individuals were less likely to have insurance. Marital status distribution also varied according to geographic area of residency, the Northwest, Northeast, and North Central areas of Florida had a larger percentage of divorced individuals, while the Central West and Southeast areas of Florida had a higher proportion of single patients. Central and Southwest Florida were most commonly comprised of married patients, whereas widowed individuals narrowly surpassed divorced individuals in the Central East region. Divorced and widowed patients were more likely to be current and former tobacco users, respectively, whereas single patients were less likely to be former users. Widowed patients were more likely to be diagnosed with lentigo malignant melanoma, while a larger percentage of single patients were diagnosed with superficial spreading melanoma and nodular melanoma. The percentage of patients diagnosed at an early stage was higher for married and lower for single patients.

The demographic characteristics associated with an increased proportion of deceased patients in this sample were older age, widowed marital status, male sex, African-American race, Hispanic ethnicity, former tobacco use, and Central East Florida geographical location (Table 2). Melanoma-related characteristics including nodular melanoma type, later stage at diagnosis, and melanoma originating on the skin of the scalp or neck were also associated with an increased proportion of deceased patients (Table 2).

**Figure 1:**
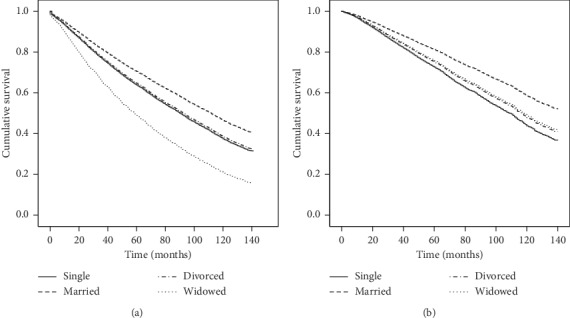
Mantel–Cox survival analysis for Florida melanoma patients between 2001 and 2009 by marital status: single (solid line), married (dashed line), divorced (dashed and dotted line), and widowed (dotted line) patients. (a) The unadjusted cumulative survival in months after diagnosis. (b) Comparison of the adjusted cumulative survival in months after diagnosis. The curves are adjusted for age at diagnosis, sex, insurance status, race, ethnicity, tobacco use, tumor histology, tumor staging, primary site, and geographic area.

The median survival time (and 95% CI) for married, divorced, single, and widowed patients was 111 (108–114), 100 (92–108), 93 (86–100), and 59 (56–62) months, respectively (log-rank test *P* < 0.001).  Figure 1 shows the curves for the unadjusted (Figure 1(a)) and adjusted (Figure 1(b)) Mantel–Cox survival analysis in months after diagnosis amongst single (solid), married (dashed), divorced (dashed and dotted), and widowed (dotted) patients. After adjusting for age at diagnosis, sex, insurance status, race, ethnicity, tobacco use, histology, staging, primary site, and geographic area, married patients were more likely to survive (HR = 0.65; 99% CI 0.57 to 0.74; *P* < 0.001) than single patients (Table 3, Figure 1). Other characteristics independently associated with lower survival were age (for every year after diagnosis, the hazard of dying increased by 5% HR = 1.05; 99% CI 1.04 to 1.05; *P* < 0.001), male sex, black race, Hispanic ethnicity, Northwest Florida geographic area, former or current tobacco use, and nonsuperficial melanoma subtype. Additionally, late staging increased the risk of death by over 300% compared with early stages. Finally, the presence of melanoma on the lower limb and hip was associated with significantly reduced risk of mortality.

Results were robust after further adjustment for histologic subtype. Results were more significant for all marital status groups when controlling for five factor 2000 SEER staging, so dichotomizing the staging as an early (in situ and localized) and late (regional by direct extension, regional lymph nodes, and distant) stage is conservative to the results. Lastly, stratified analysis and test for interaction comparing regression models with or without interaction terms suggested no evidence for effect of modification by gender in the association between marital status and melanoma survival (*P* value for overall interaction = 0.189); thus, results for both genders were presented combined.

## 4. Discussion

Our findings suggest that married individuals diagnosed with melanoma have a 35% lower risk of death than single individuals. Those who were divorced or widowed did not have a statistically different risk of death than those who were single after adjusting for confounders.

Previous studies have shown an association between marital status and stage of diagnosis and also found health benefits associated with marriage. For instance, a retrospective cohort study of 734,889 patients using the Surveillance, Epidemiology, and End Results Program (SEER) registries from 2004 through 2008 for various types of cancer (melanoma excluded) found married patients were 17% less likely to present with metastatic disease, had 53% higher odds of receiving a definitive therapy, and were 20% less likely to die as a result of their cancer than unmarried patients [12]. Our study has been built upon previous studies by showing a significant association between marital status and improved survival in melanoma patients after controlling for tumor stage at diagnosis. Therefore, it is possible that earlier detection does not fully explain improved survival in married patients. Other factors such as social support may also play a role in survival, as suggested by others [17].

Our results fail to find evidence for an effect modification by gender. This contrasts with previous studies that reported that married men enjoyed an improved survival, but that married women did not [5, 8, 18]. Married women, like married men, experience increased social support relative to their nonmarried counterparts, and so observing an increased survival in both sexes makes sense. The reasons for the discrepancy between our study and prior studies are unclear, but the difference may be related to cultural differences in the unique population of Florida. Additionally, there is some evidence that increased social support and feelings of wellbeing may bolster patients' immune systems [19] against melanoma, a very immunogenic malignancy [20].

We incidentally found that selected geographic areas in Florida had significantly decreased death rates compared with the Northwest Florida. Potential explanations for these findings might relate to differences in the availability of both family physicians and dermatologists and higher melanoma mortality in geographic locations with lower availability of physicians as previously reported [21]. Lastly, we found a significantly higher survival in people that developed melanoma in the primary sites of the lower limb and hip relative to the face. This incidental finding is yet to be confirmed and calls for further investigation.

Selected limitations require consideration when interpreting these findings. We were unable to control for factors previously described to affect survival in melanoma patients such as patient comorbidities, individual-level socioeconomic status, education level, and body mass index [8, 22]. Married individuals have previously shown to have higher BMI and higher levels of comorbidities compared with single individuals [23, 24]. Thus, lack of adjustment for these factors is likely to underestimate the association between married patients and survival. However, residual confounding through variables such as socioeconomic status is very likely and may overestimate the association. Therefore, it is not possible to determine the direction or magnitude of uncontrolled confounders' impact on the true association. Also, marital status was assessed at the time of diagnosis, thus change in marital status during the follow-up was not accounted for. Yet, evidence from previous studies regarding cancer survival fail to find an association between diagnosis of cancer and divorce rate [25]. In addition, we believe that, in our study sample, changes in marital status following a cancer diagnosis is most likely from married to divorced or widowed, rather than from nonmarried to married. In this scenario, our findings most likely represent an underestimation of the survival advantage for married patients compared with single patients. The present study was limited because cause of death was not available in the FCDS, so cause specific mortality could not be established. Although overall survival remains a robust measure of mortality, overall survival also leaves an opportunity for confounding variables to affect the outcome of death through alternative pathways. Finally, we cannot exclude the possibility that our findings are due to lead time bias, where earlier detection of melanoma occurred in married people while having no improvement of survival.

Other notable limitations involving the data include staging and histology. Ideally, melanoma staging data would include precise American Joint Committee on Cancer (AJCC) staging and Breslow depth; however, available staging information was limited to SEER staging. Nonetheless, our findings were robust after controlling for available stage at diagnosis. The majority of the patients in the data had their histological subtype listed as melanoma, not otherwise specified. Therefore, since precise information on melanoma histological subtype was unavailable for a relatively large percentage of the data, histology as a known confounding variable could not be fully controlled.

## 5. Conclusions

The present study is one of the largest and most comprehensive studies of patients with malignant melanomas reported in the state of Florida; it has an average follow-up of 10 years and it accounts for various patient demographics and melanoma characteristics. We found evidence that married patients, both men and women, are more likely to survive after a diagnosis of melanoma compared with single patients. Future studies that assess the potential mechanisms by which marriage confers survival advantages to melanoma patients are warranted as they might guide development of interventions aimed at improving survival in single patients who are diagnosed with melanoma.

## Figures and Tables

**Table 1:** Characteristics of Florida melanoma patients between 2001 and 2009 by marital status.

		Total *N* = 36,578 (100%)	Marital status				*P* value
			Single *N* = 5,037 (13.8%)	Married *N* = 25,438 (69.5%)	Divorced *N* = 2,443 (6.7%)	Widowed *N* = 3,660 (10.0%)	
Age at diagnosis^*∗*^	Mean (SD)	62.5 (16.2)	51.0 (19.2)	62.9 (14.5)	59.0 (13.1)	78.0 (9.9)	<0.001

Sex	Male	58.7	57.1	64.0	45.4	32.7	<0.001
	Female	41.3	42.9	36.0	54.6	67.3	

Insurance status	No insurance	3.4	8.9	2.4	6.4	1.3	<0.001
	Insurance	96.6	91.1	97.6	93.6	98.7	

Race	White	98.9	98.3	99.1	99.2	99.0	<0.001
	Black	0.6	0.8	0.5	0.5	0.8	
	Other	0.5	0.9	0.4	0.3	0.2	

Ethnicity	Non-Hispanic	96.6	95.8	96.9	95.0	96.9	<0.001
	Hispanic	3.4	4.2	3.1	5.0	3.1	

Geographic area	Northwest	4.9	4.8	4.9	5.8	3.9	<0.001
	Northeast	6.5	6.0	6.6	7.2	5.5	
	North central	3.6	3.8	3.6	4.6	3.1	
	Central west	20.9	22.7	20.5	20.1	21.8	
	Central	18	15.0	19.3	15.5	14.7	
	Central east	10	8.6	10.0	11.0	11.1	
	Southeast	27.7	32.4	26.0	29.5	31.8	
	Southwest	8.5	6.7	9.2	6.3	8.2	

Tobacco use	Never	61.8	63.3	61.9	54.6	63.6	<0.001
	Current	13.6	21.2	11.8	22.9	9.3	
	Former	24.6	15.5	26.3	22.5	27.1	

Histology	Acral lentiginous	0.8	0.8	0.7	0.9	1.1	<0.001
	Lentigo maligna	6.5	4.1	6.6	5.6	9.3	
	Superficial spreading	12.1	13.6	12.3	12.0	9.2	
	Nodular melanoma	5.3	6.9	4.9	5.7	6.0	
	Other	75.3	74.6	75.6	75.7	74.4	

Staging	Early	88.5	84.3	89.9	85.0	86.0	<0.001
	Late	11.5	15.7	10.1	15.0	14.0	

Primary site	Face	15.0	11.7	15.0	12.0	21.2	<0.001
	External ear	3.1	2.5	3.3	2.3	3.3	
	Scalp and neck	7.9	7.5	8.3	5.8	6.9	
	Trunk	31.5	35.2	32.0	33.2	21.7	
	Upper limb and shoulder	25.9	24.8	25.8	27.6	27.0	
	Lower limb and hip	16.7	18.4	15.6	19.2	19.9	

SD = standard deviation; results are presented as % using the survey design unless otherwise specified. ^*∗*^ANOVA; all others, chi-square test. The races grouped under “others” consist of Micronesian, Chamorro/Chamoru, American Indian, Aleutia, Alaskan Native or Eskimo (includes all indigenous populations of the Western hemisphere), Chinese, Polynesian, Japanese, Tahitian, Filipino, Samoan, Hawaiian, Tongan, Korean, Melanesian, Vietnamese, New Guinean, Laotian, other Asian, including Asian, not otherwise specified, Hmong, Pacific Islander, Kampuchean, Thai, Asian Indian or Pakistani, Asian Indian, Pakistani, Guamanian, Fiji Islanders, and Oriental, not otherwise specified, as well as unknown races. The histological categories grouped under “others” consist of Malignant Melanoma NOS, Balloon Cell Melanoma, Regressing Melanoma, Meningeal Melanomatosis, Amelanotic Melanoma, Melan Junction Nevus, Melan in Precanc Melanoma, Desmoplastic Melanoma, Mucosal Lentiginous Melan, Melan Giant Pig Nevus, Epithelioid/Spindle Cell Melan, Epithel Cell Melanoma, Spindle Cell Melanoma NOS, Spindle Cell Melanoma type A, Spindle Cell Melanoma type B, and Blue Nevus Malignant.

**Table 2:** Characteristics of Florida melanoma patients between 2001 and 2009 by vital status at last contact.

		Vital status		*P* value
		Dead *N* = 9,384 (25.7%)	Alive *N* = 27,194 (74.3%)	
Age at diagnosis	Mean (SD)	71.57 (14.2)	59.4 (15.6)	<0.001

Marital status	Single (never married)	23.3	76.7	<0.001
	Married	23.3	76.7	
	Divorced	26.2	73.8	
	Widowed	45.2	54.8	

Sex	Male	30.3	69.7	<0.001
	Female	19.1	80.9	

Insurance status	No insurance	24.8	75.2	0.786
	Insurance	25.2	74.8	

Race	White	26.0	74.0	<0.001
	Black	36.7	63.3	
	Other	16.9	83.1	

Ethnicity	Non-Hispanic	25.8	74.2	0.006
	Hispanic	29.4	70.6	

Geographic area	Northwest	25.8	74.2	<0.001
	Northeast	26.2	73.8	
	North central	21.7	78.3	
	Central west	26.1	73.9	
	Central	22.4	77.6	
	Central east	29.1	70.9	
	Southeast	26.3	73.7	
	Southwest	26.3	73.7	

Tobacco use	Never	22.6	77.4	<0.001
	Current	29.9	70.1	
	Former	35.2	64.8	

Histology	Acral lentiginous	33.7	66.3	<0.001
	Lentigo maligna	24.4	75.6	
	Superficial spreading	15.7	84.3	
	Nodular melanoma	43.6	56.4	
	Other	26.0	74.0	

Staging	Early	18.9	81.1	<0.001
	Late	61.1	38.9	

Primary site	Face	28.2	71.8	<0.001
	External ear	29.9	70.1	
	Scalp and neck	32.1	67.9	
	Trunk	21.3	78.7	
	Upper limb and shoulder	21.9	78.1	
	Lower limb and hip	19.2	80.8	

SD = standard deviation. Results are presented as % using survey design unless otherwise specified. The races grouped under “others” consist of Micronesian, Chamorro/Chamoru, American Indian, Aleutia, Alaskan Native or Eskimo (includes all indigenous populations of the Western hemisphere), Chinese, Polynesian, Japanese, Tahitian, Filipino, Samoan, Hawaiian, Tongan, Korean, Melanesian, Vietnamese, New Guinean, Laotian, Other Asian, including Asian, not otherwise specified, Hmong, Pacific Islander, Kampuchean, Thai, Asian Indian or Pakistani, Asian Indian, Pakistani, Guamanian, Fiji Islanders, and Oriental, not otherwise specified, as well as unknown races. Histological categories grouped under “others” consist of Malignant Melanoma NOS, Balloon Cell Melanoma, Regressing Melanoma, Meningeal Melanomatosis, Amelanotic Melanoma, Melan Junction Nevus, Melan in Precanc Melanoma, Desmoplastic Melanoma, Mucosal Lentiginous Melan, Melan Giant Pig Nevus, Epithelioid/Spindle Cell Melan, Epithel Cell Melanoma, Spindle Cell Melanoma NOS, Spindle Cell Melanoma type A, Spindle Cell Melanoma type B, and Blue Nevus Malignant.

**Table 3:** Associations between marital status and survival in Florida melanoma patients between 2001 and 2009.

		Unadjusted		Adjusted	
		HR (99% CI)	*P* value	HR (99% CI)	*P* value
Marital status	Single (never married)	Reference	Reference	Reference	Reference
	Married	0.78 (0.72–0.85)	<0.001	0.65 (0.57–0.74)	<0.001
	Divorced	0.97 (0.86–1.10)	0.566	0.89 (0.74–1.08)	0.117
	Widowed	1.59 (1.44–1.76)	<0.001	0.87 (0.74–1.01)	0.019
Age at diagnosis		1.04 (1.04–1.04)	<0.001	1.05 (1.04–1.05)	<0.001

Sex	Female	Reference	Reference	Reference	Reference
	Male	1.49 (1.40–1.58)	<0.001	1.33 (1.22–1.46)	<0.001

Insurance status	Insurance	Reference	Reference	Reference	Reference
	No insurance	1.36 (1.16–1.58)	<0.001	1.76 (1.42–2.19)	<0.001

Race	White	Reference	Reference	Reference	Reference
	Black	1.63 (1.21–2.20)	<0.001	1.66 (1.10–2.52)	0.002
	Other	0.83 (0.51–1.35)	0.315	0.99 (0.51–1.93)	0.966

Ethnicity	Non-Hispanic	Reference	Reference	Reference	Reference
	Hispanic	1.29 (1.12–1.49)	<0.001	1.39 (1.13–1.71)	<0.001

Tobacco use	Never	Reference	Reference	Reference	Reference
	Current	1.33 (1.21–1.46)	<0.001	1.461 (1.304–1.638)	<0.001
	Former	1.36 (1.26–1.46)	<0.001	1.14 (1.05–1.25)	<0.001

Histology	Superficial spreading	Reference	Reference	Reference	Reference
	Acral lentiginous	1.38 (1.19–1.60)	<0.001	1.10 (0.91–1.34)	0.182
	Lentigo maligna	2.22 (1.68–2.95)	<0.001	1.30 (0.91–1.86)	0.055
	Nodular	2.75 (2.41–3.14)	<0.001	1.56 (1.33–1.83)	<0.001
	Other	1.54 (1.39–1.71)	<0.001	1.10 (0.97–1.25)	0.046

Staging	Early	Reference	Reference	Reference	Reference
	Late	4.32 (4.05–4.60)	<0.001	3.49 (3.18–3.82)	<0.001

Primary site	Face	Reference	Reference	Reference	Reference
	External ear	1.06 (0.91–1.24)	0.333	0.97 (0.78–1.19)	0.664
	Scalp and neck	1.18 (1.06–1.32)	<0.001	1.09 (0.94–1.26)	0.142
	Trunk	0.80 (0.73–0.87)	<0.001	1.00 (0.89–1.13)	0.989
	Upper limb and shoulder	0.82 (0.75–0.90)	<0.001	0.91 (0.80–1.02)	0.034
	Lower limb and hip	0.75 (0.68–0.83)	<0.001	0.85 (0.74–0.98)	0.004

Geographic area	Northwest	Reference	Reference	Reference	Reference
	Northeast	0.70 (0.60–0.82)	<0.001	0.66 (0.53–0.83)	<0.001
	North central	0.72 (0.59–0.88)	<0.001	0.75 (0.56–0.99)	0.009
	Central west	0.77 (0.68–0.88)	<0.001	0.68 (0.56–0.84)	<0.001
	Central	0.54 (0.47–0.62)	<0.001	0.57 (0.47–0.70)	<0.001
	Central east	0.88 (0.76–1.02)	0.025	0.77 (0.62–0.96)	0.002
	Southeast	0.75 (0.66–0.85)	<0.001	0.68 (0.56–0.83)	<0.001
	Southwest	0.77 (0.67–0.90)	<0.001	0.67 (0.53–0.84)	<0.001

SD = standard deviation; ref = reference; HR = hazard ratio; CI = confidence interval. The races grouped under “others” consist of Micronesian, Chamorro/Chamoru, American Indian, Aleutia, Alaskan Native or Eskimo (includes all indigenous populations of the Western hemisphere), Chinese, Polynesian, Japanese, Tahitian, Filipino, Samoan, Hawaiian, Tongan, Korean, Melanesian, Vietnamese, New Guinean, Laotian, Other Asian, including Asian, not otherwise specified, Hmong, Pacific Islander, Kampuchean, Thai, Asian Indian or Pakistani, Asian Indian, Pakistani, Guamanian, Fiji Islanders, and Oriental, not otherwise specified, as well as unknown races. The histological categories grouped under “others” consist of Malignant Melanoma NOS, Balloon Cell Melanoma, Regressing Melanoma, Meningeal Melanomatosis, Amelanotic Melanoma, Melan Junction Nevus, Melan in Precanc Melanoma, Desmoplastic Melanoma, Mucosal Lentiginous Melan, Melan Giant Pig Nevus, Epithelioid/Spindle Cell Melan, Epithel Cell Melanoma, Spindle Cell Melanoma NOS, Spindle Cell Melanoma type A, Spindle Cell Melanoma type B, and Blue Nevus Malignant.

## Data Availability

The datasets generated and/or analyzed during the current study are available in the Florida Cancer Data System (FCDS)[13] repository upon request, http://www.floridahealth.gov/diseases-and-conditions/cancer/cancer-registry/index.html.

## Abbreviations

FCDS: Florida Cancer Data System
SEER: Surveillance, epidemiology, and end results program
AJCC: American Joint Committee on Cancer
CI: Confidence interval
HR: Hazard ratio
SD: Standard deviation
SPSS: Statistical package for the social sciences
US: United States.
